# Effect of including a dietary supplement of raisins, a food rich in polyphenols, on cognitive function in healthy older adults; a study protocol for a randomized clinical trial

**DOI:** 10.1186/s12877-023-03882-6

**Published:** 2023-03-29

**Authors:** María J. Rodrigo-Gonzalo, José I. Recio-Rodríguez, Roberto Méndez-Sánchez, María C. Pablos-Hernández, Alfonso González-Ramírez, Jesús González-Sánchez, María J. Fermoso-Palmero, Ana S. Puente-González, María C. Sánchez-Sánchez, Fausto J. Barbero-Iglesias, María I. Rihuete-Galve, Susana González-Manzano

**Affiliations:** 1 Grupo de Investigación de Polifenoles, Universidad de Salamanca, 37007 Salamanca, Spain; 2 Facultad de Enfermería Y Fisioterapia, Universidad de Salamanca, 37007 Salamanca, Spain; 3 Unidad de Investigación en Atención Primaria de Salamanca (APISAL), Instituto de Investigación Biomédica de Salamanca (IBSAL), Red de Investigación en Cronicidad, Atención Primaria Y Promoción de La Salud (RICAPPS), 37007 Salamanca, Spain; 4 Grupo de Fisioterapia, recuperación funcional y ejercicio terapéutico del, Instituto de Investigación Biomédica de Salamanca (IBSAL), 37007 Salamanca, Spain; 5 Hospital Universitario de Salamanca, Universidad de Salamanca, 37007 Salmanca, Spain; 6 Escuela de Enfermería de Zamora, Universidad de Salamanca, 49022 Zamora, Spain

**Keywords:** Cognitive performance, Polyphenols, Raisins, Older adults

## Abstract

**Background:**

Polyphenols have been shown to be effective against many chronic diseases, including neurodegenerative diseases. Specifically, the consumption of raisins, being a food rich in polyphenols, has been attributed with neuroprotective benefits. Therefore, our main objective is to evaluate the effect of including 50 g of raisins in the diet daily for 6 months, on the improvement of cognitive performance, cardiovascular risk factors and markers of inflammation in a population of older adults without cognitive impairment.

**Methods:**

Design and intervention: This study will be a randomized controlled clinical trial of two parallel groups. Each subject included in the study will be randomly assigned to one of two study groups: control group (no supplement), intervention group (50 g of raisins daily during 6 months).

Study population: The participants will be selected by consecutive sampling in the Primary Care consultations of urban health centers in Salamanca and Zamora (Spain), taking into account the selection criteria.

Study variables: Two visits will be made, baseline and at 6 months. Cognitive performance will be evaluated (Mini-Mental State Examination test, Rey Auditory Verbal Learning Test, verbal fluency and montreal cognitive assessment (Moca)). It will also be analyzed the level of physical activity, quality of life, activities of daily living, energy and nutritional composition of the diet, body composition, blood pressure, heart rate, markers of inflammation and other laboratory tests of clinical relevance (glycaemia, total cholesterol, HDL cholesterol, LDL cholesterol and triglycerides). In addition, sociodemographic data, personal and family history, medication use and alcohol and tobacco consumption will be collected.

**Discussion:**

In this project, it is intended to contribute to minimize the problems derived from cognitive deterioration in older people.

**Trial registration:**

ClincalTrials.gov Identifier: NCT04966455 Registration date: July 1, 2021.

## Background

According to the World Health Organziation, between 2015 and 2050 the world population over 60 years of age will increase from 900 million to 2 billion, which represents an increase of 12 to 22% [1]. Due to the increase in longevity, the prevalence of neurodegenerative diseases and consequently cognitive impairment and dementia, are increasing exponentially in recent years.

Dementia is not part of normal aging, it is a disease that involves the deterioration of memory, intellect, behavior and the ability to perform activities of daily living. It is one of the main causes of disability and dependency among the elderly, being overwhelming both for those who suffer from it and for their caregivers and relatives. It is estimated that between 5 and 8% of the population over 60 years of age will suffer from dementia at some point in time [2]. The increasing incidence of the disease and the lack of curative pharmacological treatments justify the demand for prevention strategies.

Evidence suggests that certain diets are associated with a lower incidence of neurodegenerative diseases, so maintaining a healthy diet may impact many risk factors for cognitive decline. A model to follow seems to be the Mediterranean diet [3]. The Mediterranean dietary pattern includes as distinctive characteristics, a daily consumption of vegetables, fruits, whole grains and healthy fats; the moderate intake of red wine and the use of extra virgin olive oil; all of them foods with a high content of phenolic compounds.

Phenolic compounds are a group of secondary plant metabolites, synthesized through the shikimate/phenylpropanoid and acetate/malonate pathways, characterized by having a structure with at least one phenol unit. Based on their chemical structures, they can be divided into different subgroups, such as phenolic acids, flavonoids, tannins, coumarins, lignans, quinones, stilbenes, and curcuminoids. Among them, flavonoids constitute one of the largest groups and are widely distributed in the human diet. Despite the great variety of existing flavonoids, their wide distribution and the important variations in their content, it is considered that only a limited number of these compounds are present in significant amounts in the foods that are commonly consumed in the diet. These include compounds derived from three anthocyanidins (cyanidin, delphinidin, malvidin), three flavan-3-ols (catechin, epicatechin, epigallocatechin), three flavonols (quercetin, kaempferol, myricetin), two flavanones (hesperetin, naringerin), two flavones ( apigenin, luteolin) and three chalcones [4].

During the last decade, there has been a growing interest in the possible cognitive benefits of consuming various subclasses of polyphenols, such as the flavanol and stilben family. These are present in a large number of plant-based foods, such as tea, cocoa, grapes, and red wine [5–7]. One concept to keep in mind is that polyphenol extracts used in supplementation and fortification may lack the synergistic effects on the health benefits of a diet naturally rich in polyphenols [8].

The mechanisms exerted by food polyphenols in their biological activity for the prevention and treatment of brain disorders are still under discussion, but it is believed that they may be due to direct mechanisms, possibly counteracting neuroinflammation and oxidative stress, thus improving the memory and/or cognitive function, or they may be due to indirect mechanisms that involve, among others, the modulation of the microbiota-gut-brain axis [9–11].

Neuroprotective benefits have been attributed to moderate consumption of grape polyphenols [12]. In fact, grapes are one of the richest sources of polyphenols. A serving of unpeeled grapes (200 g) could provide up to 72 mg of total phenols (Flame Seedless), including anthocyanins (cyanidin, malvidin, peonidin, petunidin, and delphinidin), the main phenolic compounds in red grapes; flavan-3-ols (catechin, epicatechin, procyanidins B1, B2 and C1, and gallocatechin), which are the most abundant phenolics in white varieties, and to a lesser extent derivatives of hydroxybenzoic and hydroxycinnamic acids, stilbenes (resveratrol) and flavonols (especially rutinoside and quercetin glycoside) present in both red and white grapes [13]. This diversity of compounds makes grapes, and especially red grapes, excellent candidates for testing the role of dietary polyphenols in health.

The consumption of raisins dates back to prehistoric times, in which the grapes were dried for storage and transport, and there is evidence of the early use of raisins as a food. In the Mediterranean region it has been a frequently consumed food and is one of the dry fruits that characterize the Mediterranean diet [14]. The phenolic profile of raisins differs from that of grapes, due to hydrolysis and oxidation processes that occur in these compounds.

Clinical research related to the possible health benefits of raisins has intensified over the past 10 years. Raisins have been reported to have a low to moderate glycemic index and a low insulin index [15]. In an intervention study with raisin ingestion for 12 weeks, a significant reduction in blood pressure and increased satiety was observed, through leptin and ghrelin, which have been related to a reduction in cholesterol values, from low density lipoproteins, triglycerides and oxidized fatty acids in serum, as well as proinflammatory cytokines [16]. Therefore, raisins could have an effect in significantly reducing the risk of developing diabetes or cardiovascular disease. However, no studies have been found in which the effect of raisins on cognitive impairment in older people has been studied. More research is required to establish the effects at the cognitive level and the molecular mechanisms by which these effects would occur.

## Methods/design

### Objectives

According to the background, the main objective of this study is to evaluate the effect of an intervention based on including 50 g of raisins in the diet daily for 6 months, on the improvement of cognitive performance, cardiovascular risk factors and markers of inflammation in a population of older adults without cognitive impairment.

### Design

**Fig. 1:**
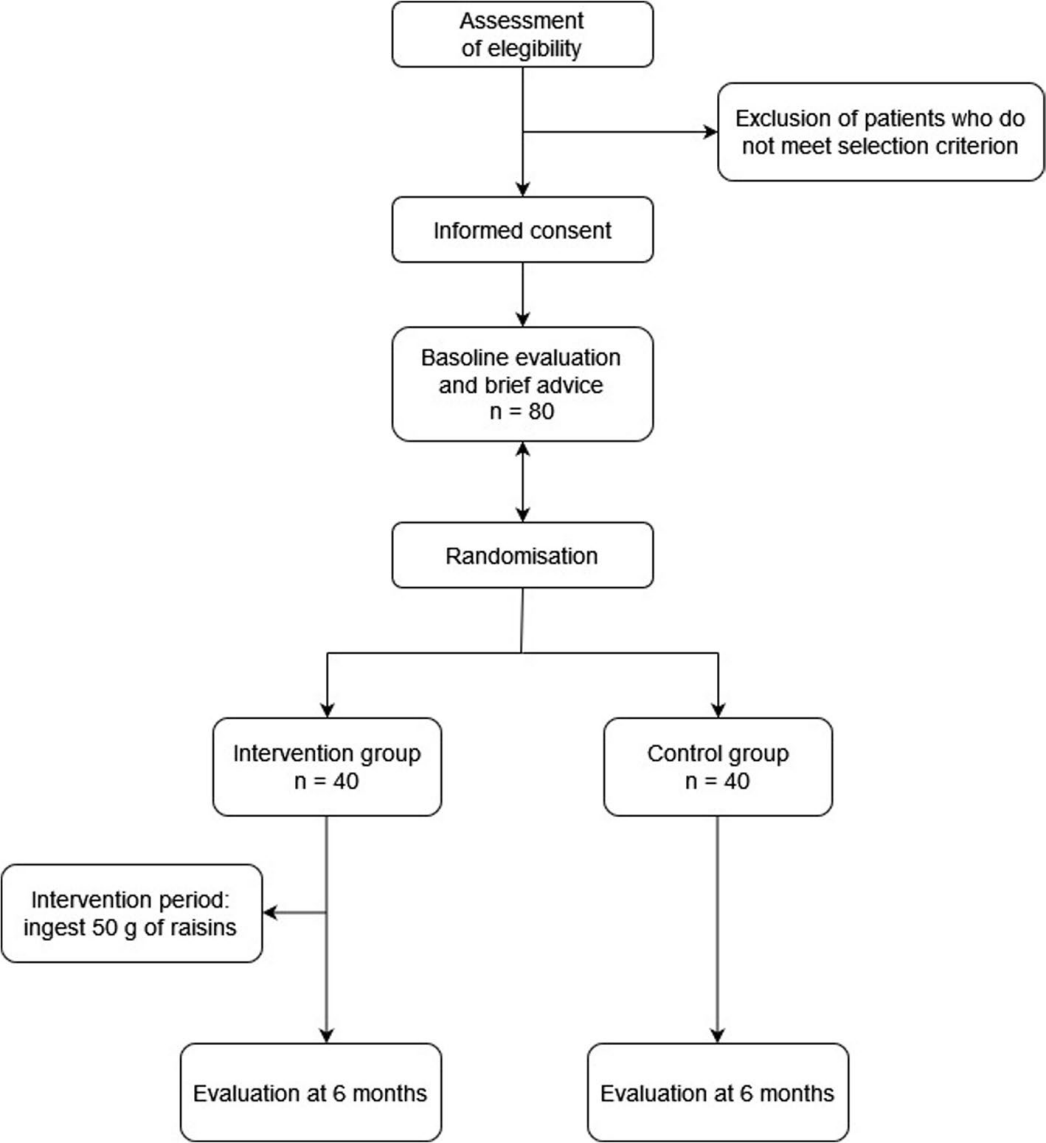
Study flow-diagram

This study will be a randomized controlled clinical trial of two parallel groups. Each subject included in the study will be randomly assigned to one of two study groups: control group (no supplement), intervention group (50 g of raisins daily). The duration of the intervention will be 6 months. Detailed in Fig. 1.

### Study population

The participants will be selected by consecutive sampling, in the Primary Care consultations of urban health centers in Salamanca and Zamora (Spain). Each Primary Care team (doctor or nurse), taking into account the inclusion and exclusion criteria, will propose the patients to participate in the study. In case they are interested in participating, they will collect their personal data and deliver it to the main researcher. Previously, the main researcher will give a clinical session with the Primary Care team to explain the characteristics of the research study. The study population will include a sample of 80 people.

### Inclusion criteria

People of both sexes over 70 years of age, Mini Mental State Examination (MMSE) score greater than or equal to 24 points, autonomous to attend the center where the study is carried out and sign the informed consent.

### Exclusion criteria

Coronary or cerebrovascular atherosclerotic disease, diabetes mellitus, heart failure grade II or higher, moderate or severe chronic obstructive pulmonary disease, advanced kidney or liver disease, severe mental illness, oncological disease under treatment diagnosed in the last 5 years or terminal condition, morbid obesity (BMI ≥ 40 kg/m2), intolerance and/or allergy to any of the components of grapes or any other circumstance at the discretion of the researchers.

### Sample size

The sample size was estimated taking into account the main variable of the study, the change in cognitive performance (categorical fluency). Accepting an alpha risk of 0.05 and a beta risk of 0.2 in a bilateral contrast, 40 subjects in the intervention group and 40 in the control group are needed to detect a difference equal to or greater than 2 points in the categorical fluency test. The common standard deviation is assumed to be 4.75 and a correlation coefficient between the initial and final measurement of 0.8. These estimates have been made based on contrasted data in the study by Garcia-Yu et al. [17]. Losses to follow-up of 10% have been estimated.

### Recruitment

The assignment of participants to the intervention group (IG) or control group (CG). The allocation sequence will be generated by an independent researcher using the Epidat 4.2 program (Department of Health, Government of Galicia, Spain), with a ratio of 1/1. The assignment will remain hidden until the participants are assigned to each group.

### Study variables

The main variable of the study will be the cognitive performance measured by several tests. The secondary variables will be body composition, blood pressure, heart rate, level of physical activity, quality of life, activities of daily living, energy and nutritional composition of the diet, markers of inflammation and other laboratory tests. clinical relevance.

### Methods used in the evaluation of cognitive performance

#### Mini Mental State Examination (MMSE) [18]

This test will evaluate temporal and spatial orientation, fixation, attention and calculation capacity, memory, nomination, repetition, comprehension, reading, writing and the drawing. The score ranges between 0 and 30 points, considering that a score greater than 24 represents normal cognitive performance.

#### Rey Auditory Verbal Learning Test [19]

This test analyzes memory retention and immediate recall, verbal learning of a list of words, and the subject's retention capacity after a non-amnestic interference task. The validated version in Spanish will be used.

#### Verbal fluency [20]

It is a categorical fluency test in which the participant is asked to list animals for one minute.

#### Montreal cognitive assessment (Moca*) *[21]

With this cognitive battery, short-term memory, visuospatial capacity, executive functions, attention, concentration, working memory, language and temporal-spatial orientation will be evaluated. The validated version in Spanish will be used. The total score is 30 points, with ≥ 26 points being an indicator of normality. If the subject has less than 12 years of schooling, a point will be added to the score obtained when taking the test.

### Methods used in the evaluation of secondary variables

#### International Physical Activity Questionnaire (IPAQ) [22]

The short version will be used, which consists of 7 questions about the frequency, duration and intensity of the activity (moderate and intense) carried out during the last 7 days. It also evaluates the time spent walking and the time spent sitting.

#### Quality of life instrument of the World Health Organization (WHOQOL) called WHOQOL-AGE [23]

Which has been specially adapted to the elderly population. This short version has 13 of the 100 questions of the original version and has been validated in populations older than 50 years. Results can range from 0 (lowest quality of life) to 100 (highest quality of life).

#### EuroQol 5-D Questionnaire [24]

It will be used to assess health-related quality of life. We will use an adapted version of this questionnaire that has been validated for the Spanish population. This questionnaire consists of three elements: the assessment of the individual's health status in severity levels by dimensions (mobility, self-care, usual activities, pain/discomfort and anxiety/depression), the assessment of their health status on a visual analog scale and, finally, an index of social values obtained for each state of health generated by the instrument.

#### Functional Activity Questionnaire (FAQ)

It was designed by Pfeffer and is based on a previous study by the same autor [25, 26]. Assesses the ability to perform complex social activities, also known as instrumental activities of daily living. There are 11 items: managing money, shopping, making tea or coffee and turning off the appliance, preparing food, keeping abreast of community events, understanding and discussing radio and television news, reading magazines and books, remembering important dates, managing medication, traveling alone outside the neighborhood, greeting friends, and going out alone safely. Each item scores between 0 (normal) and 3 (totally dependent).

#### Dietary record for 3 days (one of them on the weekend) to know the individual intake [27]

Participants should write down the food and drinks eaten on each day, being as precise as possible. To facilitate said registration, a visual guide of food rations will be delivered and explained. Through the computer program "Food and health" (Institute of Nutrition and Food Technology of the University of Granada, Spain) the energy expenditure at rest, daily caloric needs, caloric intake, carbohydrates, proteins, lipids, fatty acids monounsaturated, polyunsaturated and saturated, fiber, vitamin B6, vitamin B12, vitamin D, folic acid, potassium, calcium, iron and iodine will be estimated. In addition to the portions of fruit and vegetables and vegetables during the 3 days.

#### Markers of inflammation

Interleukin 6 (IL-6), interleukin 1 (IL-1), interleukin 10 (IL-10), C-reactive protein and tumor necrosis factor (TNF-α) will be determined. (Thermo Fisher Scientific, 88–7066-88, 88–7261-22, 88–7106-22, KHA0031, KHC3011 respectively). The ELISA technique will be used to determine serum levels in serum samples obtained at visits. The blood will be collected in refrigerated tubes containing EDTA and the samples will be centrifuged at 2000 rpm at 4ºC. The sample will be separated into aliquots of approximately 0.5 ml for each determination in order to avoid thawing, freezing and the possible degradation of the sample proteins. Subsequently, they will be stored at -80ºC until the test is carried out.

#### Indicators of cardiovascular risk

Blood pressure and heart rate will be measured with the digital blood pressure monitor model M6 Omron Comfort (Omron Healthcare, Co., Ltd. Japan) validated according to the protocol of the European Society of Hypertension [28]. Anthropometric variables will also be measured, body weight with the clinically validated Omron BF511 body composition meter [29], height with the Seca 213 portable stadiometer with integrated leveler, and hip and waist circumference with a flexible tape measure.

### Intervention

#### Common intervention in both groups

All participants (control and intervention), before random assignment is made, and after the baseline visit, will receive brief nutritional advice (3 min) from one of the principal investigators, aimed at maintaining a balanced diet.

#### Specific intervention in the experimental group

Instructions will be given for the daily intake, in addition to the usual diet, of 50 g of raisins. The duration of this intervention will be 6 months. The variety of raisins for the study has been selected after analyzing the phenolic profile of 5 types of raisins (Corintio, Moscatel Argentina, Moscatel Málaga, Moscatel Chile and Sultanas). The raisin grape selected for the study was the Moscatel Málaga grape. The composition of polyphenols per 100 g of raisins is 1.98 mg of hydroxycinnamic acids, 13.49 mg of flavanols and 4.47 mg of flavonols. This represents a total of 19.47 mg of polyphenols per 100 g of Moscatel Málaga grape. The daily nutritional contribution of 50 g of grapes would be: 150 kcal; 0.25 g fat; 36 g of carbohydrates of which 14 g are from sugars; 1 g protein; 0.02 g of salt). Participants in this group will receive as many raisins as they need until the next replenishment visit.

### Structure and planning of visits

The principal investigators will contact the possible candidates recruited in the consultations of the health centers to participate in the study, and will arrange an interview in which the objective of the study will be explained to each participant, inviting them to sign the informed consent in case that they agree to participate. Each participant will have 2 visits: a baseline visit at the beginning of the study and another visit 6 months after the baseline visit. The duration of each visit will be approximately 90 min.

#### Baseline visit

Once the fulfillment of the selection criteria in each participant has been reviewed, the initial data will be collected, which will include sociodemographic data, personal and family history, use of medications and consumption of alcohol and tobacco. A physical examination will be performed and a plasma sample will be drawn to determine the laboratory data for the study. In addition, cognitive performance, physical activity, activities of daily living and quality of life will be evaluated. They will be given and explained how to make a dietary record for 3 days that they will later deliver. At the end of the baseline visit, random assignment will be made to one of the two study groups. People included in the intervention group will be given the amount of raisins that they will add to their usual diet for a month (1500 g per month). To facilitate the recording of the consumption of the raisins, each participant included in the intervention group will be given a diary/calendar in which they will write down the daily intake of the raisins (day and time). The control group will not receive any amount of raisins.

#### Resupply visits

They will only be performed in the intervention group, for the sole purpose of replenishing the raisins. During these visits, the raisin intake diary/calendar for the previous month will be collected and a new one will be delivered along with the corresponding raisins. This task will be carried out by a researcher who is not involved in the initial and 6-month interviews.

#### Evaluation visits at 6-months

They will be carried out 6 months after the first visit. The same variables will be collected as in the baseline visit except for sociodemographic data, already collected in the first visit.

### Statistic analysis

The results will be expressed as the mean ± standard deviation in quantitative variables or by frequency distribution in the case of qualitative variables. Normality will be evaluated using the Kolmogorov–Smirnov Test. In cases where a normal distribution cannot be assumed, the corresponding non-parametric tests will be used. The chi square test will be used to analyze the association between independent qualitative variables and the McNemar test for paired samples. Using the Student's t-test (Mann–Whitney U) for independent samples, the means between two groups will be compared, evaluating the change within the same group with the Student's t-test for paired data (Wilcoxon's test). The relationship between quantitative variables will be analyzed using Pearson's correlation coefficient (Spearman's correlation coefficient). A multivariate analysis will be carried out using different multiple linear and logistic regression models to analyze the effect of adding 50 g of raisins to the usual diet on the improvement of cognitive performance, on the modification of weight and body composition, blood pressure, glycaemia, in the improvement of the quality of life and changes in the markers of inflammation.

In all cases, it will be adjusted taking into account the variables that may influence the result, such as sociodemographic variables, those related to lifestyles (physical activity and diet, smoking status), drug use, the presence of comorbidity or the basal BMI, among others. For bilateral hypothesis tests, an alpha risk of 0.05 is set as the limit of statistical significance. The statistical program will be SPSS, v.26.0.

## Discussion

The aging of the population can be considered a success for public health policies and socioeconomic development, but it also constitutes a challenge for society, which has to achieve an adequate functional capacity for this group of people with different actions. In Spain, the population pyramid continues its aging process, with a progressive increase in the proportion of people over 65 years of age. Neurodegenerative diseases are very common in the group of older people. There is no doubt about the physical, psychological and social impact it has on both the patient and their families. In addition to the social, economic and health cost that these diseases entail.

In this project, it is intended to contribute, with preventive strategies, to minimize the problems derived from cognitive deterioration in older people.

One of the most important environmental factors in the prevention of chronic diseases is adherence to a balanced diet from the beginning of life. According to scientific evidence, following adequate dietary patterns can have a great impact on the prevention and/or delay of neurodegenerative diseases. This accumulated knowledge could contribute to changing and improving the standards or criteria for planning and evaluating balanced diets, and recommending them at a healthcare level, potentially benefiting millions of people. The challenge would be to establish dietary strategies (intake of polyphenols, among others) to prevent or delay cognitive decline and thus improve the quality of life and well-being of the population. Our proposal is expected to contribute to elucidating the role of polyphenols in the relationship between diet and health.

The research group plans to publish the results of this project in high-impact international journals of different specialties with the intention of guaranteeing maximum visibility. A first publication of the primary results will be made and then another publication with the secondary results. In addition, the results will be disseminated on social networks and other media. It is intended to link the project with the completion of a Doctoral Thesis by compendium of articles.

The study follows all the CONSORT recommendations, but due to the nature of the intervention, the participating subjects will not be blind to the intervention.

## Data Availability

The datasets used and/or analysed during the current study are available from the corresponding author on reasonable request.

## Abbreviations

CG: Control group
CONSORT: Consolidated Standards Of Reporting Trials
EDTA: Ethylenediaminetetraacetic acid
FAQ: Functional Activity Questionnaire
HDL cholesterol: High-density lipoprotein colesterol
IG: Intervention group
IL: Interleukin
BMI: Body Mass Index
IPAQ: International Physical Activity Questionnaire
LDL cholesterol: Low-density lipoprotein colesterol
MMSE: Mini Mental State Examination test
TNF: Tumor necrosis factor
WHOQOL: World Health Organization Quality of Life Instrument
WHOQOL-AGE: World Health Organization quality of life instrument adapted to the elderly
